# Breastfeeding at discharge and in the third stage of the Kangaroo Mother Care among hospitalized preterm infants

**DOI:** 10.1590/1980-220X-REEUSP-2023-0383en

**Published:** 2024-06-17

**Authors:** Ana Carolina Silva dos Santos, Elenice Valentim Carmona, Clara Fróes de Oliveira Sanfelice, Reginaldo Roque Mafetoni, Maria Helena Baena de Moraes Lopes, Talita Balaminut

**Affiliations:** 1 Universidade Estadual de Campinas, Faculdade de Enfermagem, Campinas, SP, Brazil.

**Keywords:** Kangaroo-Mother Care Method, Breast Feeding, Infant, Premature, Maternal and Child Health, Método Madre-Canguro, Lactancia Materna, Recien Nacido Prematuro, Salud Materno-Infantil, Método Canguru, Aleitamento Materno, Recém-Nascido Prematuro, Saúde Materno-Infantil

## Abstract

**Objective:**

To identify the type of feeding and analyze the sociodemographic and clinical factors associated with exclusive breastfeeding at hospital discharge, in the first and in the last follow-up visit of the third stage of the Kangaroo Mother Care among infants admitted to the kangaroo unit.

**Method:**

Longitudinal and retrospective study. A total of 186 infants of gestational age <37 weeks admitted to the kangaroo unit in 2018 and 2019 was included. Data collected from medical records and subjected to inferential analysis and the Poisson regression model (*P* < 0.05).

**Results:**

Exclusive breastfeeding rate was 73.1% at discharge, with a drop at the last follow-up visit (68.1%). At discharge, there was a greater probability of exclusive breastfeeding in younger mothers, with higher education, infants born with higher birth weight and who received exclusive human milk during hospitalization; in the first follow-up visit, in a younger mother and infant who received only human milk during hospitalization; and in the last follow-up visit, a young mother, infant who received only human milk and suckled at the breast for the first time in the kangaroo unit.

**Conclusion:**

Most infants hospitalized in the second stage of the Kangaroo Mother Care were exclusively breastfed and presented maternal and clinical factors related to breastfeeding. This fact can help manage the challenges of the method and promote breastfeeding.

## RESUMO

**Objetivo:**

Identificar o tipo de alimentação e analisar os fatores sociodemográficos e clínicos associados ao aleitamento exclusivo na alta hospitalar, no primeiro e no último retorno da terceira etapa do Método Canguru entre neonatos internados na unidade canguru.

**Método:**

Estudo longitudinal e retrospectivo. Incluídos 186 neonatos com idade gestacional <37 semanas admitidos na unidade canguru em 2018 e 2019. Dados coletados do prontuário submetidos à análise inferencial e ao modelo de regressão Poisson (*p* < 0,05).

**Resultados:**

Taxa de aleitamento exclusivo foi de 73,1% na alta, com queda no último retorno (68,1%). Na alta, houve maior probabilidade de aleitamento exclusivo em mãe mais jovem, com escolaridade superior, neonato nascido com maior peso e que recebeu leite humano exclusivo durante internação; no primeiro retorno, em mãe mais jovem e neonato que recebeu apenas leite humano na internação; e no último retorno, mãe jovem, neonato que recebeu apenas leite humano e com primeira sucção na mama na unidade canguru.

**Conclusão:**

A maioria dos neonatos internados na segunda etapa do Método Canguru estava em aleitamento exclusivo e apresentou fatores maternos e clínicos relacionados ao aleitamento, podendo auxiliar no manejo dos desafios do método e na promoção da amamentação.

## RESUMEN

**Objetivo:**

Identificar el tipo de alimentación y analizar los factores sociodemográficos y clínicos asociados a la lactancia materna exclusiva al alta hospitalaria, en el primero y en la última visita de seguimiento de la tercera etapa del Método Madre Canguro entre los recién nacidos ingresados en la unidad canguro.

**Método:**

Estudio longitudinal y retrospectivo. Se incluyeron 186 neonatos en edad gestacional <37 semanas ingresados en la unidad canguro en 2018 y 2019. Datos recopilados de historias clínicas sometidas a análisis inferencial y modelo de regresión de Poisson (p < 0,05).

**Resultados:**

La tasa de lactancia materna exclusiva fue del 73,1% al alta, con descenso en la última visita de seguimiento (68,1%). Al alta hubo mayor probabilidad de lactancia materna exclusiva en madres más jóvenes, con mayor escolaridad, recién nacidos con mayor peso al nacer y que recibieron leche materna exclusiva durante la internación; en la primera visita de seguimiento, en una madre más joven y un recién nacido que recibió únicamente leche materna durante la hospitalización; y en la última visita de seguimiento, una madre joven, recién nacido que recibió sólo leche humana y con la primera succión del pecho en la unidad canguro.

**Conclusión:**

La mayoría de los recién nacidos hospitalizados en la segunda etapa del Método Madre Canguro fueron amamantados exclusivamente y presentaron factores maternos y médicos relacionados con la lactancia materna, que pueden ayudar a gestionar los desafíos del método y promover la lactancia materna.

## INTRODUCTION

Annually, around 15 million infants who require hospital care in the world are preterm, low birth weight or sick, and can benefit from low-cost, evidence-based interventions to prevent morbidity and mortality^(1)^. In Brazil, the policy of Humanized Care for Low Weight Infants, the Kangaroo Mother Care (KMC), has contributed to comprehensive, qualified and humanized care for these infants and their families^(2)^.

Proposed in three stages, the first one begins with prenatal care, followed by the infant’s admission to the Neonatal Intensive Care Unit (NICU) and/or Conventional Neonatal Intermediate Care Unit (CoNIMCU), with the aim of bringing the family closer to the infant, promoting bond formation, recommending the kangaroo position as early as possible, and encouraging parental participation in the care routine. The second stage takes place in the Kangaroo Neonatal Intermediate Care Unit (KNIMCU), also called the kangaroo unit, where the mother stays continuously with her baby, allowing the practice of the kangaroo position for as long as possible, the continuity of breastfeeding (BF), and the clarification of doubts. It is when the mother takes on most of the care for her child, supported and guided by the healthcare team. The third stage begins with hospital discharge and involves specific outpatient follow-up and home follow-up shared with primary care, to monitor the child’s first weeks at home^(2)^.

Studies point to the benefits of KMC: it reduces the risk of neonatal infections; promotes weight gain^(3,4)^; regulates and improves sleep quality^(3,5)^; improves comfort after feeding in the kangaroo position compared to the prone position^(6)^; contributes to neuropsychomotor development^(4,5)^; provides greater safety for parents when caring for their baby, including after hospital discharge; reduces neonatal stress and pain, especially during painful procedures^(2,5)^; reduces hospitalization time^(7,8)^; stimulates milk production; reduces maternal stress levels^(9)^; reduces morbidity and mortality^(3)^; and reduces the human and material resource costs for the institution^(5)^.

Furthermore, KMC also promotes immediate and long-term benefits for BF among preterm and low birth weight infants and their mothers, being considered a facilitating practice for breastfeeding^(10)^ and one of the intervention proposals to encourage BF in this vulnerable population^(11)^. This method improves affection and bonding behaviors between the binomial and, consequently, stimulates breastfeeding, allowing greater frequency, precocity, and duration of BF, improving the effectiveness of the first feeding and reducing the time to obtain effective sucking^(4,10,12)^, with effective results in the maintenance of BF^(11)^.

However, adherence to KMC practices often faces material, structural, and operational difficulties, requiring continued education of the health team for the KMC stages, to implement good neonatal practices and recognize the impacts of this method in clinical practice^(13)^. Therefore, identifying exclusive breastfeeding (EBF) rates in infants experiencing KMC can be an important indicator of the quality of care provided and guide interventions to promote, protect, and support BF in this vulnerable population.

Moreover, the implementation of all stages of the KMC is still very heterogeneous in Brazilian public and private institutions, with few hospitals having KNIMCU and outpatient follow-up in the third stage. This setting limits research that fully addresses KMC, with scarcity of studies analyzing the binomial in its second stage, in the admission to a kangaroo unit, as well as its monitoring in the third stage. Thus, this research can contribute with subsidies for the implementation of the KMC in all its stages, to stimulate family bonding, the early establishment of BF, and the maintenance of EBF among newborns.

The identification of sociodemographic and clinical factors related to BF in infants from KMC can also help in managing the challenges of care, according to the specificities, demands and priorities of care, in addition to encouraging the early performance of the second and third KMC stages. Therefore, the objective of this study is to identify the type of feeding and analyze the sociodemographic and clinical factors associated with EBF at hospital discharge, in the first and in the last follow-up visit of the third stage of KMC among infants admitted to the kangaroo unit.

## METHOD

### Design of Study

This is a longitudinal, correlational, retrospective study with a quantitative approach. During the conduction of the study, the checklist for observational studies known as STrengthening the Reporting of OBservational studies in Epidemiology (STROBE)^(14)^ was used.

### Local

The study location was the KNIMCU and the neonatology outpatient clinic of a public university hospital, a reference in care for women and infants in the city of Campinas, São Paulo. The hospital has been qualified and is a national reference for Humanized Care for Low Weight Infants, KMC, since 2017, and is accredited as a Baby-Friendly Hospital. In addition to the Human Milk Bank, KNIMCU has three beds available for binomials.

### Population and Selection Criteria

The study population consisted of medical records of infants admitted to the KNIMCU of the aforementioned hospital in 2018 and 2019. Those admitted in 2020 and 2021 were not included due to the decrease in the number of admissions to the KNIMCU during this period, with the closure of one bed due to restrictions imposed by the SARS-CoV-2 virus pandemic.

Infants with a gestational age (GA) at birth of less than 37 weeks and who were staying with their mothers for at least 24 hours in the second stage of KMC were included. Infants who died during hospitalization were excluded. Those readmitted to KNIMCU were included in the sample only once, with data from the last admission to that unit.

### Sample Definition

A sample size calculation was carried out to estimate a proportion considering p = 0.5, the value of which represents the maximum variability of the binomial distribution, generating an estimate with the largest possible sample size. A finite population consisting of 207 participants (2018 and 2019) was also considered, according to hospital records, assuming a sampling error and a significance level of 5%. The sample size obtained was 135 infants admitted in 2018 and 2019. The study had a final sample of 186 infants. To define the sample, the infants’ medical records were selected by simple random sampling through a randomized draw of all records of those admitted to KNIMCU in 2018 and 2019. The software R-Project 4.0.5 was used for the draw.

### Instrument and Data Collection

The data collection instrument was developed by the last author, a researcher with expertise in BF and neonatal nursing, together with an undergraduate student and two other researchers in the area of women and infant’s health. It was constructed exclusively for this study, containing sociodemographic and clinical, maternal and neonatal variables, as well as data related to BF during hospitalization, at hospital discharge, at the first and last outpatient follow-up visit of the third stage of KMC. A pilot test was carried out with the application of the data collection instrument in three medical records of infants eligible for the research, which were not included in the final sample. The instrument proved to be suitable for collecting data, to respond to the objective of the study.

To define the type of BF upon admission to the KNIMCU, hospital discharge, and at the first and last follow-up visits in the third stage of KMC, outcomes of this research, the definition of the World Health Organization was considered^(15)^. For this study, the categorization of non-breastfeeding (Non-BF) was also defined as the provision of infant formula only and/or any breastmilk substitute as the only source of nutrition.

Data were collected from November 2021 to February 2022 (data until hospital discharge) and from September to December 2022 (data from the third stage of the KMC) by the first and last authors. The last author, researcher in the area of neonatal nursing and with experience in collecting data from medical records, trained and supervised the first author for the recruitment and collection of data, through joint collections, listing of unknown acronyms, and identification of the fields where the data to be collected were recorded in the medical record. The medical records for consultation and data collection were requested from the hospital’s Medical Records and Statistics Service, according to the previously draw performed. All collection took place on the hospital premises, with a maximum of ten medical records scheduled per day for consultation and collection.

### Data Analysis and Treatment

All collected data were entered into a *Microsoft Excel* ^®^ spreadsheet and transported to the software Statistical Analysis System (SAS), version 9.4, for analysis. To control the quality of these data, a prior descriptive analysis was carried out to identify typing errors and outliers, with analysis and correction of inconsistent data. For the distribution of sociodemographic and clinical variables, descriptive analysis was performed through the calculation of frequencies and percentages, and measures of central tendency and dispersion.

For the comparison, association, and regression analyses of EBF with sociodemographic and clinical data, the variable type of feeding/BF was dichotomized into EBF and Non-EBF (including predominant BF, BF and Non-BF). For comparisons between BF types in relation to quantitative variables, the unpaired Student’s t-test or the Mann-Whitney test was applied, according to data distribution, assessed by the Shapiro-Wilk test. To investigate the associations between the type of BF and other qualitative variables, the chi-square test or Fisher’s exact test was used.

Modified multiple Poisson regression models with robust variance were also constructed, considering the variables on the dichotomized type of BF, at hospital discharge, at the first and last follow-up visits of the third stage as dependent variables. The modified Poisson regression model is used as an alternative to traditional logistic regression. The independent variables with a p value of less than 0.20 in the association and comparison tests in at least one of the following moments were included in the model: maternal age; type of pregnancy; birth weight; infant sex; duration of hospitalization at KNIMCU; first suck on the maternal breast at KNIMCU; and type of milk administered during hospitalization at KNIMCU. The variables GA and birth weight and total duration of hospitalization showed multicollinearity in the three moments, with only the variable birth weight being included in the model. The variables maternal education and type of delivery were also included in the model, as they are related to breastfeeding in local clinical practice. The prevalence ratio estimates obtained were presented, as well as their respective Confidence Intervals and p values. For all analyses, a significance level of 5% was considered.

### Ethical Aspects

The study complies with Resolution no. 466/12, and was approved by the Research Ethics Committee of the Universidade Estadual de Campinas, under Opinion no. 5.027.540 in 2021, with approval of an amendment expanding data collection to the third stage of the KMC, under Opinion no. 5.578.991 in 2022. The Free and Informed Consent Form was not required as the infants included were assisted in the years 2018 and 2019 and, at the time of data collection, were no longer being monitored in the hospital’s outpatient clinic, making it difficult to locate their guardians for consent. The researchers in charge signed the Data Use Commitment Term.

## RESULTS

The medical records of 186 infants admitted to KNIMCU in 2018 and 2019 were analyzed. Of these, 35 were twins, totaling 151 mothers, of which 46.4% (70) were first-time mothers. Infants and their mothers’ characterization is described in Table 1.

**Table 1:** Distribution of sociodemographic and clinical data of infants (n = 186) and their mothers (n = 151) admitted to the kangaroo unit – Campinas, SP, Brazil, 2021–2022.

Variable	Mean (SD)	Min-Max	n	%
Maternal age (years)	27.2 (7.2)	14–43		
Maternal education (n = 138)				
Elementary education (finished and unfinished)			44	31.9
High school (finished and unfinished)			78	56.5
Higher education (finished and unfinished)			16	11.6
Marital status (n = 146)				
With a partner			103	70.5
Without a partner			43	29.5
Underwent prenatal care				
Yes			148	98.0
No			3	2.0
Number of prenatal consultations	7.1 (2.8)	0–15		
Complication during pregnancy				
Yes			143	94.7
No			8	5.3
Type of delivery				
Cesarean section			100	66.2
Vaginal/vaginal with forceps			51	33.8
Sex				
Male			93	50.0
Female			93	50.0
GA at birth (weeks)	33.3 (2.2)	26.1–36.9		
Birth weight (grams)	1768.9 (399.5)	750.0–2585.0		
Presence of pathology upon admission				
Yes			182	97.9
No			4	2.1
Admission to CoNIMCU				
Yes			182	97.9
No			4	2.1
Duration of stay at the CoNIMCU (days)	10.2 (9.2)	1–48		
Admission to KNIMC				
Yes			123	66.1
No			63	33.9
Duration of stay at the NICU (days)	8.5 (11.8)	1–60		
Duration of stay at the KNIMCU (days)	9.9 (6.0)	2–29		
Total duration of hospitalization (days)	25.6 (17.2)	4–92		
Corrected GA upon admission to KNIMCU (weeks)	35.4 (1.3)	31.6–38.3		
Postnatal age at KNIMCU admission (days)	14.9 (15.0)	1.6–75.0		
Weight upon admission to KNIMCU (grams)	1881.1 (261.8)	1315.0–2650.0		
Corrected GA at hospital discharge (weeks)	36.8 (1.2)	33.9–42.0		
Postnatal age at hospital discharge (days)	24.9 (17.2)	4–91		
Weight at hospital discharge (grams)	2082.7 (248.7)	1585.0–2745.0		
Corrected GA in the first follow-up visit (weeks)	37.4 (1.5)	34.6–44.3		
Postnatal age at first follow-up visit (days)	29.2 (18.7)	10–106		
Weight at first follow-up visit (grams)	2195.0 (315.9)	1630.0–3555.0		

SD: standard deviation; GA: gestational age; CoNIMCU: Conventional Neonatal Intermediate Care Unit; NICU: Neonatal Intensive Care Unit; KNIMCU: Kangaroo Neonatal Intermediate Care Unit.

Most (93.6%; 174) infants were discharged home from the KNIMCU, but 6.4% (12) returned to conventional care before discharge. Thus, 17 (9.13%) binomials did not return to the third stage. An average of 3.6 (±2.2) follow-up visits take place in the third stage for each binomial, with a maximum of ten consultations. The average number of days between discharge and first follow-up visit was four (±7) days and a median of two days, ranging from one to 71 days.

Regarding BF, of the mothers who had other previous pregnancies, 67.9% (55) had previous experience with breastfeeding. Only one infant had skin-to-skin contact at birth and was breastfed in the first hour of life, with 73.0% (135) not requiring resuscitation at birth. Only 12.9% (24) of the neonates suckled at the breast for the first time in the kangaroo unit, given that 87.1% (162) had already suckled at the breast prior to admission to this unit. Data on BF are described in Table 2.

**Table 2:** Distribution of breastfeeding data for neonates admitted to the kangaroo unit during hospitalization, at hospital discharge, and in the third stage of the Kangaroo Mother Care – Campinas, SP, Brazil, 2021–2022.

Variables	n	%
Type of milk administered upon admission to the kangaroo unit (n = 186)
Human/breast milk	73	39.2
Human/breast milk + infant formula	112	60.2
Infant formula	1	0.6
Type of milk administered during hospitalization in the kangaroo unit (n = 186)
Human/breast milk	67	36.0
Human/breast milk + infant formula	119	64.0
Type of feeding at hospital discharge (n = 186)
Exclusive breastfeeding	136	73.1
Breastfeeding/mixed breastfeeding	44	23.7
Non-breastfeeding	6	3.2
Type of feeding in the 1st follow-up visit of the third stage (n = 169)
Exclusive breastfeeding	125	74.0
Breastfeeding/mixed breastfeeding	40	23.7
Non-breastfeeding	4	2.4
Type of feeding in the last follow-up visit of the third stage (n = 169)		
Exclusive breastfeeding	115	68.1
Predominant breastfeeding	1	0.6
Breastfeeding/mixed breastfeeding	45	26.6
Non-breastfeeding	8	4.7

At the time of hospital discharge, a relationship was identified between BF and maternal age, the infant’s birth weight, total hospitalization time, duration of hospitalization in the kangaroo unit, and the type of milk administered during hospitalization at the KNIMCU according to bivariate analysis. Following adjustments using the Poisson regression model, there was a greater probability of EBF among younger mothers, with a higher level of education, with infants with higher birth weight and who received exclusively human milk during hospitalization at the KNIMCU, as shown in Table 3.

**Table 3:** Bivariate analysis and Poisson regression model for maternal and neonatal variables, compared to breastfeeding at hospital discharge of preterm infants admitted to the kangaroo unit – Campinas, SP, Brazil, 2021–2022.

Variables	Bivariate analysis (n = 186)			Poisson Regression (n = 173)		
	EBF	Non-EBF	p value	EBF		p value
			
	n (%)	n (%)		PR (CI 95%)	LL; UL
Maternal age	25.0 (21.0–32.0)^*^	31.0 (22.0–37.0)^*^	0.0048^‡^	0.9788	0.9659; 0.9918	0.0015
Maternal education^¶^						
Higher Education	18 (85.7)	3 (14.3)	0.2825^§^	1.3304	1.0384; 1.7047	0.0240
High school	71 (74.0)	25 (26.0)	1.0461	0.8576; 1.2762	0.6564
Elementary school	38 (67,9)	18 (32.1)	Ref.		
No. of prenatal consultations	7.0 (5.0 – 9.0)^*^	7.0 (5.0 – 9.0)^*^	0.7617^‡^			
Type of pregnancy						
Single	108 (75.0)	36 (25.0)	0.2838^§^	1.1102	0.9059; 1.3603	0.3137
Multiple	28 (66.7)	14 (33.3)	Ref.		
Type of delivery						
Cesarean section	92 (73.0)	34 (26.9)	0.9636^§^	1.1775	0.9662; 1.4351	0.1055
Vaginal	44 (73.3)	16 (26.7)	Ref.		
Prior breastfeeding^†^						
No	18 (69.2)	8 (30.8)	0.7554^§^			
Yes	50 (72.5)	19 (27.5)		
Gestational age at birth	33.9 (32.4–34.7)^*^	33.0 (31.6–34.9)^*^	0.0966^‡^			
Birth weight	1840.0 (1551.5–2065.0)^*^	1725.0 (1270.0–1940.0)^*^	0.0058^||^	1.0003	1.0001; 1.0006	0.0133
Sex						
Male	72 (77.4)	21 (22.6)	0.1858^§^	1.0428	0.8875; 1.2253	0.6109
Female	64 (68.8)	29 (31.2)	Ref.		
Total duration of hospitalization (days)	17.5 (12.0–27.0)^*^	27.5 (19.0–45.0)^*^	<0.0001^‡^			
Duration of hospitalization in the kangaroo unit (days)	8.0 (5.0–12.0)^*^	11.0 (5.0–17.0)^*^	0.0311^‡^	1.0089	0.9902; 1.0279	0.3507
First suckling on the kangaroo unit					
Yes	18 (75.0)	6 (25.0)	0.8237^§^	1.1272	0.8530; 1.4896	0.3999
No	118 (72.8)	44 (27.2)	Ref.		
Type of milk administered during hospitalization in the kangaroo unit				
Exclusive human milk	65 (97.0)	2 (3.0)	<0.0001^§^	1.6096	1.3480; 1.9219	<0.0001
Human milk and formula	71 (59.7)	48 (40.3)	Ref.		

^*^Median (interquartile range);

^‡^Mann-Whitney test;

^§^Chi-square test;

^||^Unpaired Student’s t test;

^¶^n=173 in bivariate analysis due to lack of information;

^†^ Excluding primiparous women; EBF: exclusive breastfeeding; PR: prevalence ratio; CI: Confidence Interval; LL: lower limit; UL: upper limit; Ref.: reference category.

In the first follow-up visit of the third stage of the KMC, there was a relation among BF and maternal age, the type of pregnancy, the total duration of hospitalization, and the duration of hospitalization in the KNIMCU, as well as type of milk provided during hospitalization in the kangaroo unit. With adjustments using the Poisson regression model, younger mothers and infants who received exclusively human milk during hospitalization at the KNIMCU were more likely to be on EBF at the first outpatient visit. These data are described in Table 4.

**Table 4:** Bivariate analysis and Poisson regression model for maternal and neonatal variables, compared to breastfeeding in the first follow-up visit of the third stage of the Kangaroo Mother Care among premature infants admitted to the kangaroo unit – Campinas, SP, Brazil, 2021-2022.

Variables	Bivariate analysis (n = 169)			Poisson Regression (n = 158)		
	EBF	Non-EBF	p value	EBF		p value
			
	n (%)	n (%)		PR (CI 95%)	LL; UL
Maternal age	25.0 (21.0–31.0)^*^	32.5 (28.0–37.0)	< 0.0001^‡^	0.9744	0.9619;0.9871	<0.0001
Maternal education^†^						
Higher Education	15 (71.4)	6 (28.6)	0.9526^§^	1.0545	0.7765; 1.4319	0.7340
High school	68 (74.7)	23 (25.3)	0.9798	0.8103; 1.1848	0.8334
Elementary school	34 (73.9)	12 (26.1)	Ref.		
No. of prenatal consultations	7.0 (6.0–9.0)	7.5 (6.0–9.0)^*^	0.5346^‡^			
Type of pregnancy						
Single	102 (77.9)	29 (22.1)	0.0320^§^	1.1989	0.9544; 1.5062	0.1191
Multiple	23 (60.5)	15 (39.5)	Ref.		
Type of delivery						
Cesarean section	86 (74.1)	30 (25.9)	0.9394^§^	1.1494	0.9548;1.3836	0.1414
Vaginal	39 (73.6)	14 (26.4)	Ref.		
Prior breastfeeding^**^						
No	17 (70.8)	7 (29.2)	0.9743^§^			
Yes	42 (71.2)	17 (28.8)		
Gestational age at birth	33.6 (32.3–34.6)^*^	33.3 (31.6–34.9)^*^	0.6348^‡^			
Birth weight	1810.0 (1525.0–2050.0)^*^	1732.5 (1382.5–2030.0)^*^	0.1643^||^	1.0002	0.9999; 1.0005	0.1665
Sex						
Male	67 (77.9)	19 (22.1)	0.2345^§^	1.0080	0.8566; 1.1864	0.9229
Female	58 (69.9)	25 (30.1)	Ref.		
Total duration of hospitalization (days)	19.0 (13.0–28.0)^*^	26.5 (17.5–43.5)^*^	0.0019^‡^			
Duration of hospitalization in the kangaroo unit (days)	8.0 (6.0–12.0)^*^	12.5 (7.0–18.0)^*^	0.0114^‡^	0.9952	0.9752;1.0155	0.6391
First suckling on the kangaroo unit					
Yes	14 (82.3)	3 (17.7)	0.5639^¶^	1.3251	0.9881;1.7770	0.0601
No	111 (73.0)	41 (27.0)	Ref.		
Type of milk administered during hospitalization in the kangaroo unit				
Exclusive human milk	62 (96.9)	2 (3.1)	<0.0001^§^	1.5297	1.2761; 1.8338	<0.0001
Human milk and formula	63 (60.0)	42 (40.0)	Ref.		

^*^Median (interquartile range);

^‡^Mann-Whitney test;

^§^Chi-square test;

^||^Unpaired Student’s t test;

^¶^Fisher’s exact test;

^†^n=158 in the first follow-up visit due to lack of information;

^**^ Excluding primiparous women; EBF: exclusive breastfeeding; PR: prevalence ratio; CI: Confidence Interval; LL: lower limit; UL: upper limit; Ref.: reference category.

Breastfeeding, at the time of the last outpatient follow-up visit in the third stage, according to bivariate analysis, showed statistically significant relation with maternal age, sex, total duration of hospitalization, duration of hospitalization in the kangaroo unit and the type of milk administered during hospitalization at the KNIMCU. In the adjusted multiple model, younger mothers, and infants receiving only human milk at the KNIMCU and who had their first breast suck during the period in the kangaroo unit had a higher probability of EBF, with statistical significance, according to data presented in Table 5.

**Table 5:** Bivariate analysis and Poisson regression model for maternal and neonatal variables, compared to exclusive breastfeeding in the last follow-up visit of the third stage of the Kangaroo Mother Care among preterm infants admitted to the kangaroo unit – Campinas, SP, Brazil, 2021–2022.

Variables	Bivariate analysis (n = 169)			Poisson Regression (n = 158)		
	EBF	Non-EBF	p value	EBF		p value
			
	n (%)	n (%)		PR (CI 95%)	LL; UL
Maternal age	25.0 (20.0–32.0)^*^	30.5 (24.0–34.0)^*^	0.0074^‡^	0.9836	0.9698; 0.9978	0.0235
Maternal education^†^						
Higher Education	15 (71.4)	6 (28.57)	0.9448^§^	1.1041	0.7895; 1.5440	0.5627
High school	62 (68.1)	29 (31.9)	0.9801	0.7834; 1.2261	0.8602
Elementary school	31 (67.4)	15 (32.6)	Ref.		
No. of prenatal consultations	7.0 (6.0–9.0)^*^	7.0 (5.0–9.0)^*^	0.6552^‡^			
Type of pregnancy						
Single	93 (71.0)	38 (29.0)	0.1274^§^	1.0957	0.8326; 1.4418	0.5143
Multiple	22 (57.9)	16 (42.1)	Ref.		
Type of delivery						
Cesarean section	79 (68.1)	37 (31.9)	0.9815^§^	1.0948	0.8721; 1.3742	0.4350
Vaginal	36 (67.9)	17 (32.1)	Ref.		
Prior breastfeeding^**^						
No	15 (62.5)	9 (37.5)	0.4392^§^			
Yes	42 (71.2)	17 (28.8)		
Gestational age at birth	33.9 (32.3–34.7)^*^	33.4 (31.6–34.9)^*^	0.4529^‡^			
Birth weight	1835.0 (1525.0–2055.0)^*^	1732.5 (1395.0–2040.0)^*^	0.2393^¶^	1.0001	0.9998; 1.0004	0.5404
Sex						
Male	66 (76.7)	20 (23.2)	0.0136^§^	1.2257	0.9934; 1.5124	0.0577
Female	49 (59.0)	34 (40.1)	Ref.		
Total duration of hospitalization (days)	19.0 (13.0–27.0)^*^	25.0 (15.0–43.0)^*^	0.0049^‡^			
Duration of hospitalization in the kangaroo unit (days)	8.0 (5.0–12.0)^*^	11.0 (7.0–18.0)^*^	0.0133^‡^	0.9844	0.9618; 1.0075	0.1850
First suckling on the kangaroo unit					
Yes	14 (82.3)	3 (17.7)	0.1823^¶^	1.3924	1.0401; 1.8640	0.0261
No	101(66.4)	51 (33.6)	Ref.		
Type of milk administered during hospitalization in the kangaroo unit				
Exclusive human milk	56 (87.5)	8 (12.5)	<0.0001^§^	1.4021	1.1278; 1.7432	0.0023
Human milk and formula	59 (56.2)	46 (43.8)	Ref.		

*Median (interquartile range);

^‡^Mann-Whitney test;

^§^Chi-square test;

^||^Unpaired Student’s t test;

^¶^Fisher’s exact test;

^†^n = 158 in the first follow-up visit due to lack of information;

^**^ Excluding primiparous women; EBF: exclusive breastfeeding; PR: prevalence ratio; CI: Confidence Interval; LL: lower limit; UL: upper limit; Ref.: reference category.

## DISCUSSION

Infants participating in the second stage of the KMC showed satisfactory EBF rates at hospital discharge and in the third stage of the KMC, with a slight increase in the first follow-up visit and a slight decrease in the last follow-up visit, when compared to the rates found in other studies^(11,16)^. In very low birth weight premature newborns cared for at the KNIMCU of another university hospital, the EBF rate was 16.7% at hospital discharge^(11)^. When considering infants hospitalized in NICU, CoNIMCU, and KNIMCU, EBF rates at discharge are even lower, reaching 2.14% in some hospitals^(16)^.

In the first follow-up visit of the third stage of KMC, 74.0% of infants were on EBF, a higher rate than another study that found 21.7% of preterm infants on EBF in the first KMC outpatient follow-up visit^(11)^. The rate of EBF at the first follow-up visit was similar to the percentage at hospital discharge, probably due to the small time interval between these two moments, with a median of two days, meeting the recommendation of the national KMC policy, which recommends the first consultation within 48 hours after discharge^(2)^.

On the other hand, the average weight at hospital discharge still remains high, since KMC policy recommends a minimum weight of 1,600 grams as a criterion for discharge^(2)^. Therefore, the healthcare team still have difficulties and insecurities regarding the early discharge of these preterm infants^(11)^, and the progression of interventions and strategies to overcome them are required, as well as to equip and support the family for this moment.

In the last outpatient follow-up visit, despite the EBF rate decrease in relation to hospital discharge from 73.1% to 68.1% of infants, only eight were using infant formula exclusively. This decrease can be explained by the numerous challenges in maintaining EBF at home among preterm and low birth weight babies. When considering the continuity of care through the third stage of KMC, there are difficulties for the binomial during their adaptation to the new routine and insufficient professional and social support when they face doubts and difficulties about BF, which can facilitate early weaning^(17)^.

In this study, infants had an average of 3.6 consultations in the third stage of KMC, which may not have been enough to ensure maternal safety and the full establishment of EBF. The fact that 17 babies did not return after hospital discharge and that the majority of participating families did not live in the city should be taken into account, since this creates difficulties in accessing assistance in the third stage of the KMC. This emphasizes the importance of coordination between the hospital and Primary Health Care to avoid early weaning. Although recommended, this coordination is still lacking^(18)^.

However, other studies have shown a higher proportion of EBF at six months of life in babies who received KMC during hospitalization, especially in the first stage, compared to those who received conventional care^(4,19)^.

Maternal age was presented as a factor related to the EBF of the binomials who were hospitalized in the second stage of KMC in the three moments in the bivariate analysis, remaining in the regression analysis. Thus, the one-year increase in maternal age represented an average decrease of 2.12% in the probability of exclusive breastfeeding at hospital discharge, an average decrease of 2.56% at the first follow-up visit of the third stage, and an average decrease of 1.64 % in the last outpatient follow-up visit. An opposite relationship was identified in a Baby-Friendly University Hospital, showing that maternal age greater than or equal to 35 years reduced the prevalence of BF interruption within 45 days after birth by 54%, with the hypothesis that younger women interrupt breastfeeding early and more frequently due to the need to return to occupational activities^(20)^.

Studies on the relationship between maternal age and BF are still divergent. In the context of this study, the health team may have emphasized the importance of EBF for younger mothers, leading to greater adherence to this practice. Furthermore, this young audience increasingly uses new information and communication technologies, consuming, with greater frequency and ease, information and support about BF on the internet and social media^(21)^.

Mothers with higher education also indicate an average increase of 33.04% in the probability of exclusively breastfeeding their children at discharge, compared to mothers who have elementary education. On the other hand, no differences were found between mothers with elementary and higher education, and this relationship was also not maintained after hospital discharge. Lack of access to information and low education can directly influence breastfeeding success, with a 110% increase in the chance of weaning among mothers with eight years or less of education^(20)^.

Among the neonatal factors, birth weight had a relation with EBF at hospital discharge of infants who experienced the second stage of KMC, since the increase of each ten grams in birth weight represented an average increase of 0.3% in the probability of being in EBF at discharge, despite not being statistically significant in outpatient follow-up visits. The fact that preterm infants admitted to neonatal units were on BF at the time of hospital discharge was also associated with higher birth weight in a cross-sectional study^(16)^ and in the cohort with infants weighing less than or equal to 1,500 grams and/or less than 30 weeks of GA^(22)^.

Moreover, at the time of hospital discharge, the infants had a mean corrected GA of 36.8 weeks and a mean weight of 2,082.7 grams, characteristics that favor the establishment of breastfeeding due to greater physiological maturity and a greater probability of presenting rhythmic sucking, coordinated with swallowing and breathing, although the individuality and competence of each infant should be considered^(2)^.

Upon admission to KNIMUC, only one participant received infant formula exclusively, with 38.7% of infants receiving only human/breast milk, maintaining a similar percentage throughout their hospitalization in the second stage of KMC (36.0%). The relationship between the practice of KMC and breast milk intake was found in a Chinese randomized controlled study, in which binomials who made skin-to-skin contact for at least 2.5 hours a day during hospitalization in the neonatal unit, showed a higher proportion of breast milk intake than the group that did not adopt the KMC^(4)^.

Most infants who received exclusive human/breast milk at the time of admission to the KNIMCU and throughout their hospitalization in this unit were also on EBF at hospital discharge, in the first and last follow-up visit of the third stage of KMC. During hospitalization at the KNIMCU, this relationship was maintained in the regression analysis, indicating an average increase of 60.96%, 52.97% and 40.21% in the probability of being on EBF at discharge, at the first and last follow-up visit of the third stage, respectively, compared with those who ingested breast/human milk associated with infant formula during the second stage.

Despite not taking the practice of KMC and the exclusivity of BF into account, a study with preterm infants who received predominantly breast milk showed that, during hospitalization in neonatal units, they were also fed with breast milk in any proportion at hospital discharge^(16)^. Furthermore, 30 minutes of daily skin-to-skin contact in the first month of life of preterm infants led to a greater intake of breast milk, compared to those who did not benefit from the first stage of KMC^(19)^. Thus, it is believed that skin-to-skin contact, carried out in both the first and second stages, provides greater intake of breast milk during the hospitalization of preterm infants and, consequently, a greater probability of early establishment and maintenance of EBF.

In this study, 87.1% of participants sucked for the first time before admission to KNIMCU; however, it was found that the fact that the infant had suckled the mother’s breast for the first time in the kangaroo unit represented an average increase of 39.24% in the probability of being on EBF at the last follow-up visit of the third stage of KMC compared to those who did not suckle for the first time in this unit. This can be explained by the hypothesis that infants who had already been placed on the mother’s breast before admission to the KNIMCU did not do so systematically and frequently, compared to those who started sucking in the kangaroo unit. The latter had the opportunity to remain with their mothers for 24 hours and, consequently, suck more often throughout the day, allowing EBF to be established more quickly and efficiently. In a meta-analysis study, preterm and low birth weight infants who received kangaroo care started early breastfeeding, 2.6 days earlier than babies who received conventional care^(12)^.

Therefore, these results reinforce the importance and benefits of KMC, especially in the second stage, in which mother and child are together 24 hours a day at the KNIMCU in frequent skin-to-skin contact. KMC is considered an essential component in the care of low birth weight infants, presenting a positive impact on the practice and maintenance of EBF^(11)^. In addition to this care policy, the satisfactory rates of EBF found in this study may also be a reflection of the profile of the unit, which belongs to a Baby-Friendly Hospital, where there is great effort to establish and maintain breastfeeding, with multidisciplinary actions of protection, promotion, and support for BF.

A limitation of this study is the dependence on the availability and quality of institutional records, since data were retrospectively extracted from medical records. However, this study contributes with data regarding infants and their mothers who specifically benefited from the second stage of KMC, considering joint hospitalization at KNIMCU, since most studies on the subject have been investigated from the perspective of skin-to-skin contact in neonatal units in their first stage. There is a lack of studies addressing infants who benefited from hospitalization in the kangaroo unit, as this is not a reality in most hospitals that care for at-risk infants.

Therefore, the findings on the type of feeding and factors related to EBF in infants who experienced the second stage of KMC can provide support to reinforce the importance of this stage in the EBF of preterm and low birth weight babies. They can also guide strategies and interventions by the multidisciplinary team to promote, protect, and support breastfeeding in the hospital environment and in Primary Health Care, since the first weeks after discharge are considered a critical period for early weaning, in addition to helping with the challenges of performing KMC.

## CONCLUSION

Preterm infants who participated in the second stage of KMC had satisfactory EBF rates at hospital discharge and in the third stage of KMC, which reflect the benefits and impact of this policy on the establishment of breastfeeding in this vulnerable population.

The greater probability of EBF at hospital discharge was related to younger maternal age, higher maternal education, higher birth weight, and exclusive intake of human/breast milk during hospitalization at the KNIMCU. In the first follow-up visit of the third stage of KMC, the greater probability of EBF was related to younger maternal age and the infant receiving exclusive human/breast milk during hospitalization in the second stage. In the last follow-up visit, it was related to the lower maternal age, the infant taking only human/breast milk and suckling for the first time at the breast during hospitalization at the KNIMCU.

The identification of these maternal and neonatal factors related to EBF can guide interventions and strategies for the early establishment and maintenance of EBF among preterm and low birth weight infants who experience the second and third stages of KMC, in line with the guidelines of this policy, and can provide subsidies for the expansion of this practice in neonatal units.
